# Streamlining Stigma Measurement: Validation and Abridgment of the HIV Stigma Scale for Pregnant Women Living with HIV in South Africa

**DOI:** 10.1007/s10461-025-04891-9

**Published:** 2025-11-18

**Authors:** Yumei O. Chen, Maria M. Llabre, Jennifer A. Smit, Nzwakie Mosery, Norik Kirakosian, Sanelisiwe Mngomezulu, Nothando Mhlaba, C. Andres Bedoya, Amelia M. Stanton, Steven A. Safren, Christina Psaros

**Affiliations:** 1 Department of Psychology, University of Miami, Coral Gables, FL USA; 2 Department of Obstetrics and Gynaecology, Faculty of Health Sciences, University of the Witwatersrand, Durban, South Africa; 3 Department of Psychiatry, Massachusetts General Hospital, Boston, MA USA; 4 Harvard Medical School, Boston, MA USA; 5 Department of Psychological and Brain Sciences, Boston University, Boston, MA USA

**Keywords:** HIV & AIDS, Stigma, Pregnancy, Psychometrics, IRT

## Abstract

The HIV Stigma Scale (HSS) is a 40-item psychometrically sound measure capturing four domains of perceived stigma. A 25-item version has been validated in South India. However, the HSS has not been validated among pregnant women with HIV (WWH) in South Africa, a population facing significant stigma. Moreover, they could benefit from the abridged measure to be more efficiently connected to related interventions. Analyzing data from a sample of pregnant WWH (*N* = 472) recruited from an antenatal care clinic in eThekweni, Kwazulu-Natal Province, we: (1) conducted confirmatory factor analyses (CFA) to determine if the 4-factor structure of the original 40-item scale holds for the 25-item version in this sample; (2) further shortened the 25-item version using data-driven item reduction, and (3) conducted a comprehensive psychometric evaluation on the abridged version that included internal consistency and construct validity. The CFA confirmed a four-factor structure of the 25-item scale (CFI = 1.00, RMSEA = 0.03, SRMR = 0.04) in pregnant WWH. For model identification, we retained three items per factor. CFA of this 12-item version revealed model fit (CFI = 1.00, RMSEA = 0.01, SRMR = 0.03). All four subscales in the abridged scale showed satisfactory internal consistency (Cronbach’s α > 0.80) and construct validity. This is the first study validating the HSS among pregnant WWH in an HIV-endemic setting and developing a psychometrically robust 12-item version, maintaining the four-factor structure of the original measure. Clinically, the abridged HSS enables more efficient screening and reduces participant burden, which could improve reporting and early implementation of stigma reduction interventions in resource-limited settings.

## Introduction

South Africa (SA) continues to be disproportionately affected by the HIV epidemic, with an HIV prevalence of 30% among pregnant women [1]. Prevention of mother-to-child HIV transmission (PMTCT) programs have contributed to significant progress in identifying pregnant women with HIV (WWH) and linking them to care [2, 3], but disparities persist in meeting goals for viral suppression, with only half of pregnant WWH in SA having suppressed viral loads—far below the UNAIDS 95-95-95 target, which aims for 95% of persons on antiretroviral therapy (ART) to achieve viral suppression [4–6].

HIV-related stigma, defined as negative attitudes and beliefs about people with HIV (PWH), has been shown to negatively impact HIV-related health outcomes [7, 8]. Common types of stigma reported by pregnant WWH in SA include overt stigmatizing attitudes from healthcare providers about their HIV status and pregnancy status, anticipated stigma associated with status disclosure, and internalized stigma resulting in a negative self-image [9–11]. Stigmatizing experiences can influence health outcomes through mechanisms such as discrimination, psychological distress, low levels of social support, and maladaptive coping behaviors [12–16]. These psychosocial factors are compounded by gender norms and dynamics that penalize perceived promiscuity and immorality, which unfairly target WWH [17, 18]. WWH are often blamed for acquiring HIV and held responsible for the consequences. As a result, an HIV diagnosis during pregnancy may heighten WWH’s vulnerability to ART nonadherence, leading to unsuppressed viral load [19–21].

HIV-related stigma may also reduce engagement with the PMTCT cascade, which starts with identifying all pregnant WWH and concludes with HIV testing in HIV-exposed infants at 6 months [22–26]. Each step along the cascade increases the potential for unwanted disclosure of HIV status, heightening both the anticipated and experienced stigma [27–29]. Subsequently, these stigma-related concerns can lead to challenges in the uptake of and adherence to PMTCT services in resource-limited settings like SA [30–33]. Addressing HIV-related stigma is therefore critical to improving engagement in the PMTCT cascade and ensuring better health outcomes for both mothers and their infants.

The first step to effectively address HIV-related stigma is to accurately measure and understand the multifaceted nature of enacted, anticipated, and internalized stigma using psychometrically sound instruments. Among existing HIV-related stigma measures, the HIV Stigma Scale (HSS) developed by Berger and colleagues [34] has been used widely among PWH in research and clinical settings. This scale comprises 40 items that use a 4-point Likert-type response option, ranging from ‘strongly disagree’ to ‘strongly agree’. The original measure captures four distinct dimensions of perceived HIV-related stigma verified by confirmatory factor analysis: *perceived stigma*,* disclosure concerns*,* negative self-image*,* and concerns with public attitudes.* Specifically, the *perceived stigma* subscale assesses the perceived consequences of other people knowing about one’s HIV status. The *disclosure concerns* subscale assesses one’s concerns or worries about disclosing one’s HIV status. The *negative self-image* subscale assesses the presence of negative emotions towards the self, due to one’s HIV status. Lastly, the *concerns with public attitudes* subscale assesses concerns regarding public attitudes toward PWH, including worries about keeping a job and fear of discrimination.

The HSS is considered a comprehensive measure of HIV-related stigma with strong psychometric properties. A recent systematic review examined the psychometric properties of the HSS across 166 studies worldwide, of which 24 were development and/or validation studies [35]. The HSS demonstrated internal consistency, with Cronbach’s alpha values ≥ 0.70 reported in 93.2% of the studies. Validity assessments of the HSS also yielded positive results, including face and content validity assessed by stakeholders and experts [36], as well as convergent validity when correlating the scale with measures of theoretically related constructs [34, 37–39]. Furthermore, the HSS has been administered to various populations across settings comparable to the current study, including PWH attending a tertiary facility in Ghana [38], pregnant WWH in SA [40], and WWH in Indonesia [41]. Culturally adapted and abbreviated versions of the original scale have been developed and published to reflect unique population-based stigma experiences, such as an Urdu version in Pakistan, a Spanish version in urban Peru, a Swedish version, and an English version in Myanmar [36, 42–45]. These adaptations underscore the scale’s versatility and effectiveness in capturing HIV-related stigma across various demographic groups and geographical locations.

Pregnant WWH in SA face unique challenges related to HIV stigma, which have important public health implications. Although the HSS has been administered to diverse populations, including pregnant WWH in SA, prior studies did not involve formal psychometric validation of the scale, which is essential to ensure its accuracy and relevance for this specific population. Additionally, developing a concise and tailored screening tool can help address concerns, such as nonadherence to ART and hesitations about attending medical appointments. A well-adapted tool would also improve accessibility and usability in both clinical and community settings, facilitating the implementation of interventions to address stigma.

Therefore, the current study aimed to: (1) validate the 25-item HSS—including its factor structure and construct validity—in a sample of pregnant women living with HIV (WLWH) in KwaZulu-Natal, South Africa—a province with the highest HIV prevalence among pregnant women globally [1], 2) develop a psychometrically sound abridged version of the 25-item HSS using both classical test theory and item response theory, to reduce respondent burden within a lengthy baseline survey, and 3) assess the internal consistency and construct validity of the abridged HSS.

## Methods

### Participants and Procedures

We leveraged data from a cohort of pregnant WWH (*N* = 472), recruited from an antenatal care clinic in KwaZulu-Natal that was designed to assess factors associated with attrition from HIV care following the birth of a child. Data collection was conducted from June 2017 to May 2022. Participants were recruited into the study if they met the following inclusion criteria at the baseline visit: (1) age between 18 and 45 years; (2) living with HIV; (3) at 28 or more weeks of pregnancy (i.e., in the third trimester); (4) currently on antiretroviral therapy (ART); (5) fluent in English or isiZulu, and (6) able and willing to provide informed consent and permission to be contacted by researchers for repeated assessments. Interviews were typically conducted in person or over the phone on the same day consent was obtained, unless participants requested rescheduling due to limited availability in their schedules. Assessments were conducted in isiZulu, and all measures that were not readily available in isiZulu were forward- and backward-translated by study team members and piloted by the MatCH Research Unit (MRU) staff before use to ensure the translations were culturally appropriate [46]. Ethical approval was obtained for all aspects of this study from the Human Research Ethics Committee (Medical) of the University of the Witwatersrand, as well as from the MassGeneral Brigham Research Committee in the USA.

### Measures

#### Demographics

Participants reported their age, time since HIV diagnosis, and highest educational levels ranging from “None” to “Complete post-secondary.”

#### HIV Stigma

We used the 25-item abridged version of the original 40-item HSS [34], which was developed and validated by Jeyaseelan and colleges [36] and is used to assess HIV-related stigma across four domains (Personalized Stigma, Disclosure Concerns, Negative Self-Image, and Public Attitudes), along a 4-point Likert-type scale (Strongly Disagree = 0, Disagree = 1, Agree = 2, Strongly Agree = 3). A total score was computed for each participant to capture the overall stigma they had experienced.

#### Depression

The 20-item Center for Epidemiological Studies-Depression Scale (CES-D; [47]) was used to assess the frequency of depression symptoms (e.g., restless sleep, poor appetite, and feeling lonely) within the past week along a 4-point Likert-type scale ranging from 0 (“rarely or none of the time”) to 3 (“most or all of the time”). Items 4, 8, 12, and 16 were reverse scored. A total score ranging from 0 to 60 was computed for each participant to record their overall level of depression, with high scores indicating greater depressive symptoms. The scale has satisfactory internal consistency, with Cronbach’s alpha ranging from 0.85 to 0.90, and test-retest reliability ranging from 0.45 to 0.70 [47–49].

#### Coping

The Brief COPE is a 28-item, brief form of the original COPE inventory [50] that assesses both adaptive and maladaptive coping strategies in response to stressful life events. Participants were asked to rate different ways of coping with a stressful life event on a 4-point Likert scale ranging from 1 (“I have not been doing this at all”) to 4 (“I have been doing this a lot”). For each participant, three average scores were calculated for the three coping styles corresponding to different items—problem-focused coping, emotion-focused coping, and avoidant coping, with higher scores indicating a higher level of engagement in that coping style. The brief form has demonstrated satisfactory internal consistency, with α >0.6, and construct validity, evidenced by significant correlations with relevant measures, such as subject well-being and perceived stress [51, 52].

### Analyses

Data screening, preparation, and descriptive statistics were completed in IBM SPSS Statistics, Version 27.0. Confirmatory factor analysis (CFA), item response theory (IRT), and psychometric validation, including reliability and convergent validity, were completed in R version 4.3.3. Data were screened for missingness and normality assumptions. Skewness and kurtosis were calculated for each item of the 25-item HSS to determine if responses met normality assumptions [53]. The data were found to substantially deviate from normality, which is expected given the ordinal nature of the responses. Consequently, a weighted least squares estimator with a diagonal weight matrix and robust standard errors was employed.

Our approach to analysis was twofold, blending both data-driven methods and systematic evaluation to assess the quality of items on the 25-item HSS. Through multiple rounds of examination, we evaluated the representativeness and cohesiveness of each item, as well as their effectiveness in distinguishing varying levels of HIV-related stigma. We then reduced the scale to include only items of the highest overall quality. Employing this iterative data-driven approach allowed us to scrutinize each item’s contribution to the overall scale and ensure that the final selection of items was both robust and representative.

#### Phase 1. Validation of the Factor Structure

A first-order CFA was conducted to assess the fit of the 25-item HSS to the four-factor structure (Model 1) proposed in the original 40-item scale [34]. Given the high correlations observed among the factors, a single-factor CFA (Model 2) was performed to examine the potential for consolidating the four factors into a single, overarching construct—stigma, which could provide a more parsimonious representation of the data. Finally, we conducted a higher-order CFA in which the four first-order factors were modeled as indicators of a second-order latent variable (Model 3). This approach enabled us to assess whether a broader, unifying stigma construct could account for the relationships among the four dimensions. Model fit was assessed using the χ^2^ test, Comparative Fit Index (CFI), standardized root mean squared residual (SRMR), and root mean square error of approximation (RMSEA). The data were expected to have a good model fit if the χ^2^ test was non-significant, CFI value was greater than 0.95, SRMR was lower than 0.08 and RMSEA score was lower than 0.06 [54]. Model fit across different models was compared using the Satorra-Bentler scaled chi-square difference test, which adjusts the standard chi-square by dividing it by a scaling correction factor to improve its approximation under conditions of non-normality [55].

#### Phase 2. Item Reduction

**Fig. 1:**
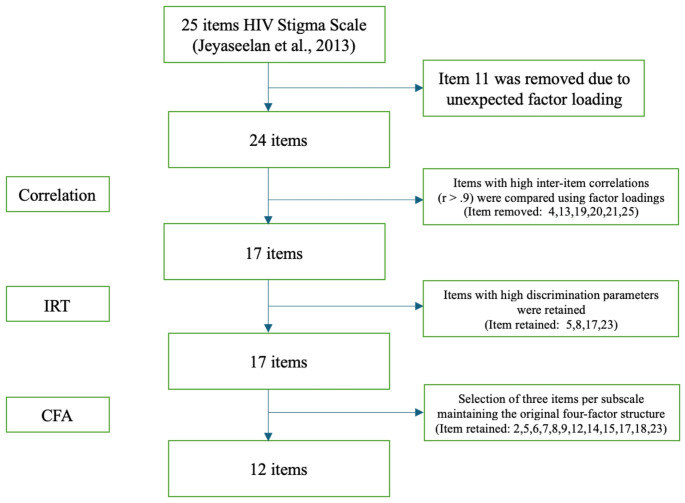
Flowchart of the item reduction process to form an abridged version of the HIV Stigma Scale

The item reduction process is shown in Fig. 1 and described in more detail below. Two primary goals guided this process: (1) to retain items with strong psychometric properties and (2) to preserve the four HIV stigma domains identified in the original scale and the 25-item version.

##### Step 1. Removing Items with Unexpected Factor Loadings

Factor loadings represent the strength and direction of the relationship between each observed item and the underlying latent construct it is intended to measure; values closer to 1 indicate a stronger association and greater contribution of the item to the latent factor. To ensure that all items uniformly measured the underlying construct after reverse coding, any item with a factor loading in the opposite direction was excluded from further consideration. This step ensured that only items consistently aligned with the construct were included.

##### Step 2. Selecting from Items with High Inter-Item Correlation

An inter-item polychoric correlation matrix (i.e., correlations between theorized continuous latent variables underlying observed ordinal responses) was generated to identify and remove potentially redundant items. The matrix allowed us to assess the relationships among items, with a specific focus on identifying highly correlated pairs. Given the range of the correlation coefficients, a cutoff threshold of 0.9 was selected to ensure the retention of the most relevant items. Within pairs exceeding this threshold, we retained the item with higher factor loading from Model 3.

##### Step 3. Using Item Response Theory to Examine Item Difficulty and Discrimination Parameters

Given the polytomous response options of the HSS and building upon the previously established unidimensionality and local independence via CFA, we fit a graded response model to our data to examine item difficulty and discrimination parameters. Item discrimination parameters indicate how well the item differentiates between respondents with different levels of the latent trait [56]. Higher discrimination values suggest that the item is more effective at distinguishing between respondents. The usual range for the discrimination parameters is from − 2 to + 2. We prioritized retaining items with the highest discrimination parameters to ensure that we preserved items that effectively distinguish between varying levels of HIV stigma experiences. On the other hand, the difficulty parameters represent the stigma level at which a respondent has a 50% chance of endorsing a particular category or higher [57]. Typically, the difficulty parameters range from − 3 to + 3 [58], where lower values indicate that the item reflects a lower level of stigma, and higher values indicate the item demonstrates a higher level of stigma.

##### Step 4. Preserving the Original Four-Factor Structure

To prevent model identification problems while maintaining the integrity of the original four-factor structure, we retained three items for each of the four dimensions [59]. The items included in the abridged version were selected based on four criteria: (a) relatively low inter-item correlation; (b) consistency with the original four-factor structure; (c) absolute magnitude of factor loadings >0.90; (d) high discrimination parameters.

#### Phase 3. Psychometric Evaluation of the Abridged Version

We conducted another second-order four-factor CFA (Model 4) to test whether the abridged version retains the factor structure of the original scale and the relationships between the first-order factors can be explained by a higher-order factor. We then evaluated the goodness of fit of Model 4 using the same model fit indices employed in the evaluation of Models 1, 2, and 3.

McDonald’s Omega was selected to measure the scale’s internal consistency, as the assumptions for Cronbach’s alpha, unidimensionality, and tau-equivalence were not met; a value greater than 0.7 was considered acceptable [60]. To assess the construct validity of the abridged version, we examined convergent validity by testing whether the abridged version and its four subscales were significantly associated with constructs known to be theoretically related to HIV stigma using Pearson’s correlation [61]. Specifically, we utilized a measure of depression and a measure of coping strategies, as prior literature has consistently demonstrated links between stigma and these concepts [62–64].

## Results

Participants were between the ages of 18 and 45 (M = 28.90, SD = 5.12). Most of the sample endorsed high school as their highest level of educational attainment (*N* = 360, 76.3%) and housing stability (*N* = 444, 94.1%). On average, participants reported 2.98 years since their diagnosis of HIV (SD = 3.32), 2.61 pregnancies (SD = 1.1), and 2.02 household members living with HIV (SD = 1.97). Complete participant characteristics are presented in Table 1.

**Table 1:** Participant (*N* = 472) characteristics

Variable	*N*	%
Educational attainment		
Less than high school	26	5.5
High school	360	76.3
Some college	56	11.9
College or more	30	6.4
Housing stability		
Yes	444	94.1
No	22	4.7
**Variable**	**M**	**SD**
Age (years)	28.90	5.12
Time since HIV Diagnosis (years)	2.98	3.32
Gravidity (number of pregnancy)	2.61	1.1
Number of household members living with HIV	2.02	1.97
Depression symptom severity	3.74	7.25
Coping	68.54	12.84
Problem-Focused Coping	3.40	0.78
Emotion-Focused Coping	2.53	0.51
Avoidant Coping	1.36	0.42
Resilience	19.75	3.47
HIV stigma	6.57	9.35

*N* Sample size, *M* Mean, *SD* Standard deviation. Depression symptom severity potential range=0–60, Coping total range=28–112 (subscale range=1–4), Resilience potential range=0–40, HIV stigma potential range=0–75

### Phase 1. Validation of the Factor Structure

**Fig. 2:**
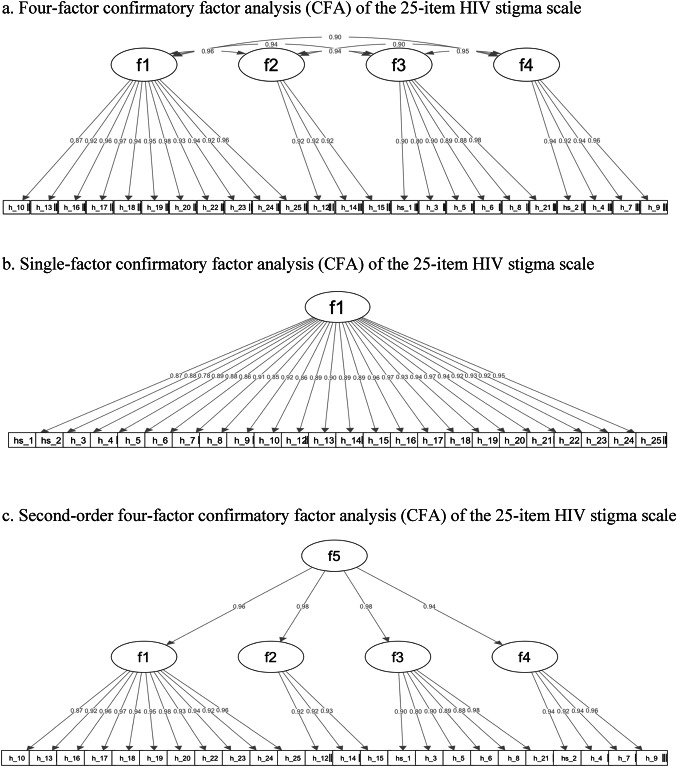
**a** Four-factor confirmatory factor analysis (CFA) of the 25-item HIV stigma scale. **b** Single-factor confirmatory factor analysis (CFA) of the 25-item HIV stigma scale. **c** Second-order four-factor confirmatory factor analysis (CFA) of the 25-item HIV stigma scale. Figures show standardized weighted least square estimates. Panel 1a presents correlations between subscales (f1: personalized stigma; f2: disclosure concerns; f3: negative self-image; f4: concerns with public attitudes) and their relationships with observed items. Panel 1b shows estimates for a one-factor model (f1: HIV-related stigma) and item loadings. Panel 1c illustrates a higher-order model with a second-order factor (f5: HIV-related stigma) loading onto the four first-order subscales, which in turn load onto their respective items

The results of the first-order four-factor CFA (Model 1) confirmed the factor structure proposed in the original study [34]. As shown in Fig. 2a, all items loaded strongly on their hypothesized four latent factors, with standardized factor loadings ranging from 0.80 to 0.98. High correlations were also observed between these first-order latent factors. The fit indices were CFI = 1.00, RMSEA = 0.03, SRMR = 0.04. Although the chi-square test was statistically significant, this result was not unexpected and did not, on its own, indicate poor model fit. The chi-square statistic is highly sensitive to sample size and model complexity, producing significant values even when models fit the data relatively well [65]. In this case, our sample size (*N* = 472) was sufficient for the chi-square test to detect even small and practically unimportant deviations between the model-implied and observed covariance matrices. Furthermore, the residuals—representing the differences between the predicted and observed covariances—remained within an acceptable range, supporting the conclusion that the model provides a good fit to the data.

Given the high correlations between first-order factors, we next tested two alternative models that offered more parsimonious representations of the data. In Model 2, we tested a single-factor model, where all items loaded onto a general stigma factor. As shown in Fig. 2b, while factor loadings remained strong (0.78–0.97.78.97), and model fit was acceptable (CFI = 1.00, RMSEA = 0.05, SRMR = 0.05), a chi-square difference test indicated that this model fit significantly worse than Model 1 (Δ χ^2^ = 85.73, *p* <.001).

In Model 3, we tested a second-order CFA to examine whether a higher-order latent construct could explain the four correlated first-order factors. As shown in Fig. 2c, all factor loadings remained high (0.80–0.98.80.98). Model fit was excellent (CFI = 1.00, RMSEA = 0.03, SRMR = 0.04). A chi-square difference test revealed that Model 3 did not fit significantly worse than Model 1(Δ χ^2^ = 13.23, *p* =.08), supporting its utility as a more parsimonious representation of the data.

Taken together, all three models demonstrate strong fit, but Model 3 offers a simplified and theoretically coherent structure and was therefore chosen to represent the data structure. Confirming the factor structure of the data informs the subsequent item reduction process, specifying that items will be reduced from each dimension rather than from the overall scale to preserve the dimensionality of the construct and ensure balanced item retention across all four stigma domains.

### Phase 2. Item Reduction

#### Step 1. Removing Items with Unexpected Factor Loadings

Item 11, “I never feel the need to hide that I have HIV,” was reverse-coded. Still, it exhibited a negative factor loading in the factor analysis, contrary to the expected positive direction, like the rest of the items. This unexpected result could mean that the respondents interpreted the item differently than intended, or that the reverse wording disrupted their usual response patterns, as this is the only reverse-worded item in the scale. Therefore, it was excluded from further item selection to maintain the scale’s internal consistency and validity.

#### Step 2. Selecting from Items with High Inter-Item Correlation

Table 2 details the inter-item correlations. Items meeting the threshold of Pearson’s *r* =.9 were further compared. For instance, Item 4 (‘most people believe that a person who has HIV is dirty’) and Item 7 (‘most people think that a person with HIV is disgusting’) had a Pearson’s r of 0.93. Item 20, “people have physically backed away from me when they learn I have HIV” was highly correlated with several items and excluded from further consideration. These items were then further compared using factor loadings from the Model 3 CFA results. Items with higher factor loadings were retained and presented in Table 3 for further analysis.

**Table 2:** Inter-item correlations for the 25-item version of the HIV stigma scale displayed by polychoric correlation matrix

	1	2	3	4	5	6	7	8	9	10	12	13	14	15	16	17	18	19	20	21	22	23	24	25
1																								
2	0.81																							
3	0.73	0.74																						
4	0.80	0.77	0.61																					
5	0.77	0.80	0.77	0.81																				
6	0.78	0.80	0.79	0.70	0.85																			
7	0.85	0.76	0.73	0.93*	0.78	0.82																		
8	0.76	0.79	0.76	0.76	0.86	0.86	0.79																	
9	0.80	0.85	0.72	0.88	0.78	0.84	0.90	0.75																
10	0.75	0.83	0.75	0.71	0.77	0.87	0.73	0.83	0.77															
12	0.84	0.81	0.67	0.83	0.83	0.70	0.81	0.75	0.81	0.72														
13	0.77	0.79	0.68	0.76	0.72	0.78	0.81	0.79	0.82	0.80	0.80													
14	0.78	0.76	0.62	0.75	0.72	0.78	0.77	0.76	0.79	0.75	0.86	0.90												
15	0.78	0.75	0.67	0.69	0.72	0.71	0.73	0.73	0.75	0.72	0.79	0.83	0.87											
16	0.79	0.83	0.73	0.79	0.82	0.77	0.79	0.76	0.86	0.81	0.83	0.83	0.80	0.86										
17	0.82	0.82	0.73	0.72	0.81	0.76	0.78	0.78	0.85	0.83	0.83	0.85	0.81	0.85	0.92*									
18	0.81	0.83	0.69	0.76	0.81	0.73	0.74	0.75	0.83	0.81	0.82	0.79	0.78	0.79	0.93*	0.92*								
19	0.75	0.82	0.71	0.80	0.82	0.73	0.82	0.78	0.85	0.76	0.83	0.84	0.78	0.88	0.92*	0.91*	0.88							
20	0.81	0.84	0.69	0.70	0.79	0.77	0.77	0.78	0.84	0.80	0.81	0.88	0.83	0.88	0.93*	0.97*	0.90	0.93*						
21	0.83	0.80	0.67	0.86	0.79	0.74	0.87	0.70	0.89	0.74	0.86	0.84	0.83	0.85	0.91*	0.90	0.87	0.91*	0.91*					
22	0.79	0.82	0.70	0.72	0.77	0.71	0.73	0.71	0.80	0.78	0.78	0.84	0.81	0.87	0.90	0.88	0.88	0.89	0.91*	0.89				
23	0.78	0.77	0.66	0.71	0.77	0.73	0.75	0.78	0.79	0.75	0.76	0.84	0.80	0.87	0.91*	0.90	0.90	0.89	0.94*	0.85	0.91*			
24	0.79	0.80	0.70	0.73	0.79	0.81	0.79	0.77	0.81	0.82	0.75	0.85	0.80	0.84	0.88	0.88	0.85	0.87	0.90	0.85	0.84	0.88		
25	0.77	0.85	0.70	0.77	0.80	0.73	0.76	0.75	0.88	0.79	0.83	0.82	0.79	0.84	0.94*	0.94*	0.90	0.93*	0.93*	0.90	0.88	0.87	0.90	

Note.*indicates correlation >0.90

**Table 3:** Item discrimination and difficulty parameters from graded response model

Item	Discrimination	Difficulty parameters		
		Threshold 1	Threshold 2	Threshold 3
1	1.28	3.00	3.72	4.39
2	1.85	2.69	3.79	4.19
3	0.95	3.32	4.43	4.46
4	0.99	1.97	2.96	3.97
5	2.32	2.73	3.53	3.57
6	1.92	2.89	4.16	4.24
7	1.01	1.87	3.02	4.59
8	2.03	2.53	3.64	3.71
9	1.00	1.93	3.31	5.13
10	1.58	3.00	4.06	4.68
12	0.80	2.54	3.11	4.49
13	0.92	2.82	3.99	4.76
14	1.05	1.74	2.97	4.29
15	0.96	3.10	4.75	5.98
16	1.74	2.79	3.82	4.93
17	3.21	2.51	3.29	3.59
18	1.95	2.77	3.66	4.56
19	1.86	2.84	4.02	4.26
20	2.84	2.80	3.88	4.48
21	1.21	1.85	2.66	3.62
22	1.31	2.80	3.99	5.14
23	2.37	2.86	4.17	4.70
24	1.56	2.87	4.01	4.44
25	1.84	2.38	2.78	3.42

#### Step 3. Selecting Items with a High Discrimination Parameter

Table 3 presents the graded response model results. Items with discrimination parameters falling outside the expected range of −2 to + 2 (i.e., Items 5, 8, 17, 20, 23) were retained for further analysis. However, Item 20 was excluded due to its high correlation with multiple items identified in Step 2. Item 18 has a discrimination parameter close to 2 but lower correlations with existing items and was thereby selected for further analysis.

Table 3 also shows a positively skewed distribution of difficulty parameters in this study, which suggests that individuals need to exhibit a relatively high level of HIV-related stigma to endorse these items consistently. Consequently, this scale may be more sensitive in detecting stigma experiences among individuals with higher levels of perceived stigma, which is aligned with the characteristics of the study population and captures subtle variations in higher levels of perceived stigma.

#### Step 4. Preserving the Original Four-Factor Structure

In the final stage of item selection, we prioritized items with the highest factor loadings based on the results of Model 3 CFA (Fig. 2c). To maintain the integrity of the four-factor structure, three items were retained within each subscale while prioritizing the inclusion of items that emerged from Steps 1 and 2. The final list of items is shown in Table 4.

**Table 4:** The 12-item abridged version of the HIV stigma scale

**Personalized Stigma**
17. People I care about stopped calling after learning I have HIV
18. Some people close to me are afraid others will reject them if it becomes known that I have HIV
23. I have lost friends by telling them I have HIV
**Disclosure Concern**
12. I worry that people may judge me when they learn I have HIV
14. I worry that people who know I have HIV will tell others
15. I regret having told some people that I have HIV
**Negative Self-Image**
5. Having HIV makes me feel unclean
6. Since learning I have HIV, I feel set apart and isolated from the rest of the world
8. Having HIV makes me feel that I’m a bad person
**Concerns with Public Attitudes**
2. People with HIV lose their jobs when employers find out
7. Most people think that a person with HIV is disgusting
9. Most people with HIV are rejected when others find out

The item numbering references the item order in the 25−item version of the HIV Stigma Scale

### Phase 3. Psychometric Evaluation of the Abridged Version

**Fig. 3:**
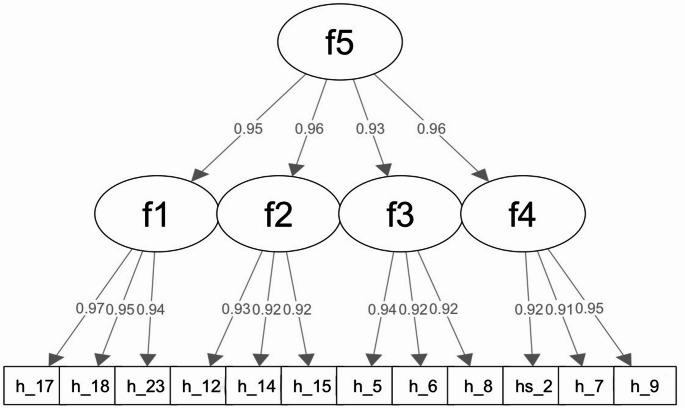
Second-order four-factor confirmatory factor analysis (CFA) of the 12-item HIV stigma scale

To test whether the proposed factor structure holds for the abridged scale, we performed a second-order four-factor CFA. As shown in Fig. 3, all model parameters were statistically significant. The standardized coefficients presented high values ranging from 0.90 to 0.98. Model fit indices showed fit, including a non-significant chi-square (χ^2^ = 48.52, df = 50, *p* =.53), CFI = 1.00, RMSEA = 0.00, SRMR = 0.03. Therefore, we concluded that the abridged scale maintained the integrity of the factor structure proposed in Model 3 and was suitable for further psychometric validation.

McDonald’s omega for the abridged scale was 0.95 and considered excellent. Similarly, the four subscales also demonstrated high reliability with omega values of 0.89, 0.86, 0.84, and 0.87, respectively. These values indicate that the items within each subscale are highly correlated and consistently measure the same underlying construct. The high omega values across both the full scale and the subscales demonstrate strong reliability and support the scale’s utility for both research and clinical applications.

Regarding construct validity, specifically convergent validity, Table 5 presents the correlations between the abridged HIV stigma scale and its subscales, as well as conceptually related measures (i.e., CES-D and Brief Cope). As hypothesized, the total stigma score and all subscale scores showed positive correlations with avoidant coping. Additionally, higher levels of depression were associated with higher total stigma scores and higher scores on the four subscales. Lastly, our results revealed a moderate negative correlation between problem-focused coping and the total stigma score, as well as the personalized stigma and disclosure concerns subscale scores, but not with negative self-image or concerns about public attitudes subscale scores. These findings suggest that the abridged scale and its subscales demonstrate acceptable construct validity.

**Table 5:** Correlations between other measures and the abridged HIV stigma scale and subscales

Variable	1	2	3	4	5	6	7	8
1. hss_new_total								
2. personalized stigma	0.833**							
3. disclosure concerns	0.906**	0.687**						
4. negative self-image	0.773**	0.550**	0.587**					
5. concerns with public attitudes	0.899**	0.673**	0.719**	0.647**				
6. cesd_total	0.533**	0.415**	0.524**	0.343**	0.498**			
7. pf_coping	− 0.097*	− 0.091*	− 0.091*	− 0.058	− 0.087	− 0.095*		
8. ef_coping	0.074	0.038	0.087	0.056	0.060	0.158**	0.736**	
9. ac_coping	0.414**	0.351**	0.363**	0.338**	0.377**	0.466**	0.146**	0.359**

**indicates *p* < .01. *N* = 472

## Discussion

This is the first study to validate the HIV Stigma Scale in a sample of pregnant WWH in KwaZulu-Natal, SA, and to produce a 12-item abridged version to streamline assessment, reduce response burden, and encourage routine screening in resource-limited settings. We employed a combined data- and theory-driven approach to retain non-redundant and cohesive items that capture stigma-related experiences within this population. Results confirmed the four-factor structure that emerged from the original validation study [34] and underscored the multifaceted yet interconnected sources of HIV-related stigma within this population. Subsequent psychometric examination of the abridged scale indicated strong reliability and construct validity, demonstrating its utility in resource-limited settings like SA. The final scale aimed to capture a wide range of stigma experiences, including an internalized sense of abandonment and relational strain, potential consequences of status disclosure, psychological ramifications of living with HIV, and the broader societal context that influences and perpetuates stigma.

The current findings have important clinical implications. The items in the final scale—selected for their high factor loadings, discriminatory ability, and minimal overlap—captured HIV-related stigma relevant to pregnant WWH in SA. This nuanced understanding may help healthcare professionals to better assess the impact of stigma and make timely referrals to appropriate interventions to address identified HIV-related stigma. Integrating psychosocial assessments into routine medical visits is often challenging, particularly in resource-limited settings like SA. Brief measures can help streamline the screening process and reduce high rates of missing data and poor reporting of patient-related outcomes, making it more feasible for clinics to adopt and implement [66, 67]. Additionally, reducing the size of assessment batteries can decrease average response time, respondent burden, and survey fatigue. Given the established impact HIV-related stigma has on health outcomes and health-related quality of life, an efficient measurement is essential and justified for routine medical visits.

This study has limitations to note. First, all the measures used in the current study were self-reported and may be subject to recall bias as well as social desirability bias, particularly given the nature of the questions. Although the item reduction process was designed to consider various factors systematically, it may have inadvertently prioritized items with the highest factor loadings, potentially overlooking nuanced aspects captured by other items. Additionally, shorter scales are generally prone to floor and ceiling effects—where a significant proportion of respondents score at the lowest or highest possible points [68]. Future studies should investigate the presence and impact of such effects in the abridged scale. Furthermore, the graded response model revealed that the scale was not effective in differentiating among individuals at the lower end of the stigma spectrum. However, this limitation should be interpreted in context. The population of interest, pregnant WWH in SA, has been consistently shown to report relatively high levels of perceived HIV stigma. Therefore, loss of measurement sensitivity at the low end of the stigma spectrum may have limited practical implications in this setting. Such a limitation could be particularly restricting in clinical or research settings where the detection of mild symptoms and the need for early interventions is critical, especially in populations that experience lower levels of stigma. Lastly, we acknowledge that examining associations with measures of related psychosocial constructs represents only one aspect of construct validity, and future work should incorporate additional methods to more comprehensively evaluate the scale’s validity.

Several avenues for future research could be pursued. First, to our knowledge, there is currently no HIV stigma scale developed specifically for pregnant WWH, despite their unique experiences related to stigma in the context of pregnancy, motherhood, and antenatal care. Further efforts could focus on developing a separate, targeted measure that captures forms of stigma most salient to this population. Additional items could also be introduced to improve the scale’s sensitivity across the full spectrum of stigma experience, such as intersectional stigma—the convergence of multiple stigmatized identities within a person. While the present study used correlations with conceptually related constructs (e.g., depression and coping strategies) to examine convergent validity, future research can employ other methods to more comprehensively examine construct validity. Additionally, future studies could examine predictive validity by assessing the ability of the scale to predict conceptually relevant outcomes, such as depression, medication adherence, and viral load, to closely monitor the impact of HIV-related stigma along the PMTCT care continuum and implement timely and stigma-informed interventions. Finally, future research could explore the key determinants of integrating the abridged scale into routine antenatal care, which may include structural and logistical factors, such as staff training requirements and the availability of psychosocial support services for referral.

In conclusion, the current study advances the assessment of HIV-related stigma among pregnant WWH in SA by validating the 25-item HSS and developing a concise 12-item version with comparable psychometric properties. The reliability and validity testing highlight its potential for efficient screening in resource-limited settings and for potentially improving access to targeted interventions. Although limitations exist, this study lays the groundwork for future studies to further refine the scale and explore other types of validity.

## Data Availability

The data that support the findings of this study are available upon reasonable request (e.g., methodologically sound proposal and signed data use agreement) to the corresponding author (C.P.), following publication.
